# Climate change and its impact on the bioactive compound profile of medicinal plants: implications for global health

**DOI:** 10.1080/15592324.2024.2419683

**Published:** 2024-10-26

**Authors:** Esther Ugo Alum

**Affiliations:** Department of Research and Publications, Kampala International University, Kampala, Uganda

**Keywords:** Climate change, medicinal plants, bioactive compounds, phytochemicals, global health

Climate change is an existential threat that has far-reaching consequences, influencing the existence of living organisms and human well-being. One of the many consequences of climate change is its impact on medicinal plants, which are scarce sources of bioactive compounds that are highly sought after in both traditional and advanced medicine. With increasing global temperatures, changes in rainfall intensity and frequency, higher levels of carbon dioxide (CO_2_), and increased occurrence of extreme weather conditions, the growth, distribution, and levels of phytochemicals in medicinal plants are changing, with implications for global health.^1^ This commentary seeks to unravel the impacts of climate change on medicinal plants, specifically on their ability to synthesize bioactive compounds and other related effects on human health. Researchers have discovered that a wide range of active compounds, such as alkaloids, flavonoids, terpenoids, and phenolics, contribute to the pharmacological activity of medicinal plants.^2^ Various factors such as temperature, light, water status, and soil type affect the production of these compounds, which are examples of secondary metabolites.^3^ By enhancing these environmental factors, climate change poses a potential threat to the concentration and profile of these bioactive compounds in the plants used in medicine.^4^ On the other hand, some climatic pressures associated with climate change, such as ultraviolet radiation or water shortages, may stimulate the production of specific phytochemicals as a plant’s defense mechanism. This may also lead to increased bioactivity in certain medicinal plants, resulting in greater effectiveness in treating diseases.^5^ Conversely, the same stressors may reduce the concentration of one or more of these bioactive compounds or even change their chemical characteristics in some way that reduces the plant’s effectiveness or makes it completely inactive. For example, changes in temperature and rainfall patterns may affect the synthesis of phytochemicals required for the plant’s pharmacological properties, as well as its medicinal value.^6^

Current research indicates that environmental stressors can substantially modify the chemical characteristics of bioactive molecules in plants. Numerous abiotic variables, such as salinity, aridity, heavy metals, illumination, temperature, and soil characteristics, can affect the biosynthesis and accumulation of plant secondary metabolites.^7^ Although certain stressors may result in a general reduction of secondary metabolites, particular cases demonstrate the overexpression of advantageous compounds.^8^ Recently, Sharma^8^ reported that aloe vera showed reduced total phenolic content, reduced antioxidant activity, and heightened aloin (a potent anti-inflammatory agent) synthesis under increased soil salinity. In the same research, drought stress resulted in a reduction in flavonoids in *Vitis vinifera, Lavandula angustifolia*, and *Artemisia tridentate*.^8^ Jan and colleagues report that transcription factors such as WRKY, MYB, bZIP, bHLH, AP2/ERF, and NAC govern stress-induced alterations in plant secondary metabolites.^9^ Understanding these modifications is critical for pharmaceutical applications, sustainable agriculture, and the preservation of medicinal plant biodiversity.^7,8^

It is a well-documented fact that medicinal plants contain a broad spectrum of secondary metabolites, which have major impacts on their defense mechanisms and carry various pharmacological activities.^10,11^ Climate change can affect the synthesis and secretion of these compounds through different physiological and biochemical processes. For instance, heightened temperatures and drought conditions can create some form of stress in plants, ultimately affecting the production of flavonoids, phenolic acids, and essential oils.^12^ These changes can alter the functionality of these plants in ecological niches, as well as their use as medication sources. This means that the variation in bioactive compounds due to climate change is of significant concern. Studies show that temperature and humidity changes directly affect the quantities of primary and secondary metabolites in the plants used in traditional medicine. For instance, St. John’s Wort (*Hypericum perforatum*) demonstrated changes in the content of its active ingredients under stress conditions that might influence the plant’s pharmacological potential.^13^ In addition, other environmental stress factors compounded by increased CO_2_ levels intensify these effects and thereby alter the characteristics of plants that are medicinal.^14^ Climate change’s effects extend beyond the alteration of phytochemicals, encompassing other aspects like biodiversity loss. Climate change further exacerbates the extinction risk for medicinal plants, with projections indicating that a large portion of plant species may experience a decline in their geographic distribution.^15^ Habitat loss, excessive use, and destructive farming practices, among others, pose a threat to most medicinal plants. Climate change intensifies these threats by altering the ecosystems that support these plants, potentially leading to the extinction of species with unexplored uses.^15^ This loss is a big setback to the community, especially those that wholly depend on herbal medicine for their health needs. In many developing regions, medicinal plants are critical and vital elements of health delivery systems because they are cheap and easily accessible tools for managing various diseases.^16^ As a result, the impact of climate change becomes particularly worrisome for such population groups. Failure to obtain such plants or a decline in their efficiency due to climate change will lead to a situation where vulnerable communities cannot access important cures for diseases, thereby further worsening inequalities in the distribution of health products around the world. The economic consequences are also evident, as medicinal plants are the primary source of income for many people. The climate-induced changes in medicinal plants also pose a threat to the pharmaceutical industry. Chemicals extracted from medicinal plants are the basis for most of today’s drugs.^17^ Changes to the concentrations and efficacies of these bioactive substances could act as barriers to drug innovation and discovery, resulting in a lengthened process and increased costs in the process of introducing new drugs into the market. Furthermore, climate change has the potential to modify the geographical range of medicinal plants, thereby inducing their migration to areas with more favorable climatic conditions. While this may have the potential to expand the cultivation of medicinal plants, it also presents challenges in terms of sustainable production, land management, and preservation. Given the documented changes, it is imperative to develop and advocate for measures that can mitigate the impact of climate change on medicinal plants and ensure continued access to these resources.

Applequist et al.^18^ advised campaigns for conservation, local cultivation, sustainability training for harvesters, preservation of traditional knowledge, and quality monitoring systems to offset the impacts of climate change in medicinal plants. Furthermore, the creation of climate-resilient plant types and the adoption of sustainable agriculture approaches are critical adaptive solutions.^19^

Therefore, this commentary suggests that addressing the impacts of climate change on medicinal plants will require an all-encompassing approach. From this standpoint, conservation efforts should include the species and genus of medicinal plants, as well as the habitats they cover. This proposed approach involves promoting the adoption of sustainable collection methods, supporting the cultivation of medicinal plants in various types of land, and preserving seeds and plant species for future use. Moreover, there is a pressing need for research that specifically examines the effects of climate change on the phytochemical profile of medicinal plants. This research should aim to identify species that are capable of flourishing in contemporary climatic conditions and investigate biotechnological methods that can enhance the synthesis of these bioactive chemicals when cultivated in challenging environmental conditions. It is critical to incorporate climate resilience into the healthcare sector’s policy planning process. Governments and international organizations must recognize the importance of medicinal plants in health delivery systems and implement preventive measures to mitigate the effects of global climate change. The objectives should include promoting and preserving indigenous traditional medical systems, enhancing interactions between scientists and indigenous communities, and improving equitable access to plant resources for medicinal purposes.

In sum, climate change’s impact on the bioactive compounds synthesized by medicinal plants is a pressing worldwide health concern. Climate change may lead to variations in the therapeutic properties of these plants, thus impacting traditional medicine, the pharmaceuticals developed from them, and global healthcare. An essential aspect to acknowledge is that conservation, research, and policy should not be independent entities but rather collaborate closely to promote the future of medicinal plants and their associated advantages. Ensuring timely intervention is crucial to prevent climate change-orchestrated medicinal plant deterioration from reaching an irreversible stage and causing further damage to global health.

## Data Availability

Additional data shall be made available by the author on request.
